# Normocalcemic Parathyroid Adenoma with Brown's Tumor Maxilla: A Rare Case

**DOI:** 10.22038/IJORL.2022.62654.3155

**Published:** 2023-01

**Authors:** Aarushi Wadhawan, Nikhil Arora, Ashiya Goel, Pratik Kumar, Parmod Jangra, Aanchal Gupta

**Affiliations:** 1 *Department of Otorhinolaryngology, * *Pt B.D Sharma PGIMS, Rohtak, Haryana* *, India.*; 2 *Department of Pathology, Pt B.D Sharma PGIMS, Rohtak, Haryana, India.*

**Keywords:** Brown's tumor, Maxillary tumor, Normocalcemia, Primary Hyperparathyroidism, Parathyroid adenoma

## Abstract

**Introduction:**

Primary hyperparathyroidism due to parathyroid adenoma commonly causes raised serum calcium and focal giant cell lytic lesions in bones known as Brown’s tumors. It is more common in females in the post-menopausal age group.

**Case Report:**

We report a case of a 29-year-old female patient with Brown’s tumor maxilla in a clinical setting of normocalcemic primary hyperparathyroidism. The patient presented to us with facial and palatal swelling for which FNAC was done. Cytology revealed hemosiderin-laden macrophages suspicious for Brown’s tumor. On further imaging studies such as CT Neck, Tc99 Sestamibi scan, and other biochemical tests like parathyroid hormone assay and serum calcium level, the diagnosis of a hyperfunctioning parathyroid gland with normal calcium level was made. Parathyroidectomy was performed and parathyroid adenoma came out to be the primary pathology. On post-operative follow up there was regression of the swelling on the face and palate relieving the patient symptomatically.

**Conclusion:**

The diagnostic suspicion of primary hyperparathyroidism should be kept in mind whenever a young female presents with suspected Brown’s tumor, even with normal serum calcium levels, for appropriate management. Ours was a highly uncommon case that was a diagnostic challenge and had a successful treatment outcome. Very few such cases have been reported in the literature to date to the best of our knowledge.

## Introduction

Osteitis fibrosa cystica also known as brown’s tumor is a non-neoplastic, reactive lesion resulting from the excessive osteoclastic activity (1). Brown’s tumor is seen in 2% of people with primary hyperparathyroidism, the most common manifestation being asymptomatic hyper calcemia (2,3). Although normocalcemia and intermittent hypercalcemia are also well-known entities associated with Brown’s tumor but they are extremely rare (4). Mandible is the most common site accounting for 4% of the patients with Brown’s tumor but it can also affect the maxilla (5). 

The disease can affect any age group, but it is more common among individuals older than 50 years and is three times more common in women than in men (6). The term Brown’s tumor is derived from its characteristic macroscopic appearance of brownish material within a cystic cavity which is because of blood pigments present in hemosiderin-laden macrophages (7). Brown’s tumor of the maxilla is an extremely rare presentation in normocalcemic primary hyperparathyroidism (8). We hereby present this uncommon case of primary hyperparathyroidism with normocalcemia with the sole manifestation of Brown’s tumor of the maxilla. After thorough literature research, we found that our case is the 2^nd^ case reported. 

## Case Report

**Fig 1:**
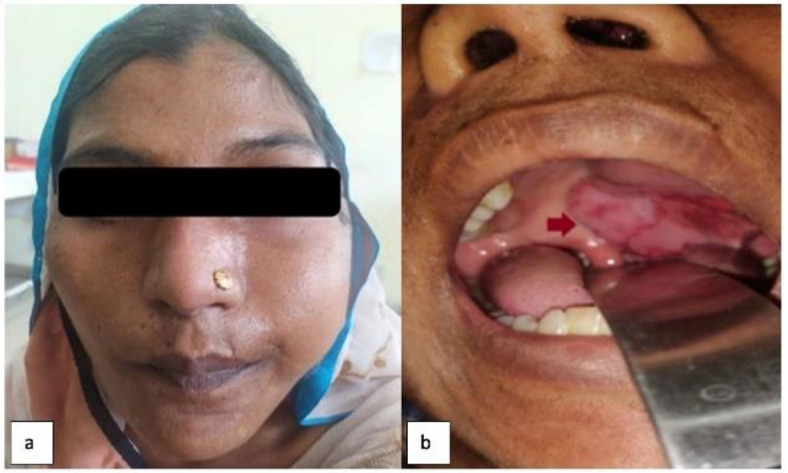
**(a) **shows diffuse facial swelling over the left cheek. **(b)** shows swelling of the left side of the palate(arrow)

A 29-year-old female came to the ENT department with complaints of swelling over her left cheek for the past 6 months (figure 1a) which was insidious in onset, progressively increasing in size, and associated with intermittent pain. Loosening of teeth was also present for the past 3 months. 

**Fig 2:**
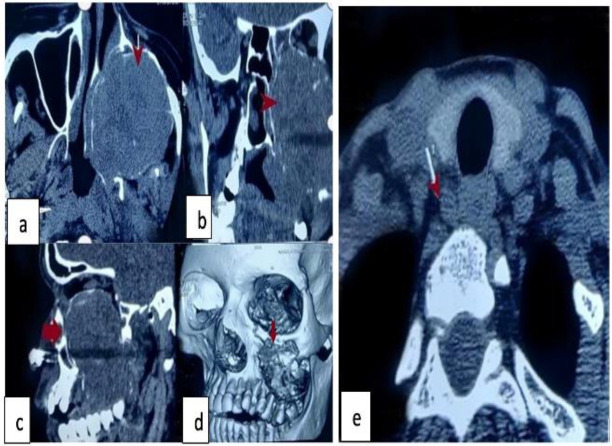
**(a) **Computed Tomography scan (axial section) showing left maxillary sinus mass(arrow) (**b,c)** CT Coronal and sagittal section with the extension of mass into palate(arrow) **(d) **3D bony reconstruction of left maxilla revealing the mass **(e) **Computed tomography of neck demonstrating enlarged parathyroid mass

A complete head and neck examination revealed a firm to hard swelling in the left cheek which was approximately 6×5cm in size. On intraoral examination- there was a soft tissue swelling involving the left half of the hard palate and extending up to the soft palate (Figure 1b). Contrast Enhanced Computed Tomographic (CECT) Scan of the Nose and Para Nasal Sinus (PNS) with 3D reconstruction (figure 2a,b,c,d) was done which revealed an osteolytic, expansile mass of approximately 6.4×5.3×7.3cm in left maxillary sinus eroding the maxillary sinus walls compressing the neighboring structures without any obvious invasion into them.

Cytological aspirate from the swelling revealed features suggestive of Brown’s tumor. Further investigations included intact parathyroid hormone assay, serum calcium and phosphate, serum alkaline phosphatase, serum vitamin D, serum creatinine, and urine creatinine. The intact parathyroid hormone serum level was found to be elevated at 297.7 (15.0-65.0 pg/mL), serum calcium level was normal-9.15 (8.6-10.3 mg/dL), serum phosphate was decreased-2.34 (3.4-4.5 mg/dl), alkaline phosphatase was elevated-360.4 (30-130 IU/L). 

Other investigations such as vitamin D, serum creatinine, and urinary calcium came out to be normal thus ruling out secondary causes of hyperparathyroidism. On ultrasonography of the neck, a hypoechoic lesion of size 18.3×9.0 mm at the inferior pole of the right thyroid lobe was reported. The patient was then posted for a CECT scan of the neck which revealed an ovoid lesion in the right trachea-oesophageal groove along the posteroinferior aspect of the right thyroid lobe likely a parathyroid lesion (figure 2e). The scan was also suggestive of Brown’s tumors like lesion in the C7 and T1 vertebrae. A Technetium 99 sestamibi (Tc 99 MIBI) scan recorded normal thyroid with increased focal tracer uptake in the right inferior pole of the thyroid. 

**Fig 3:**
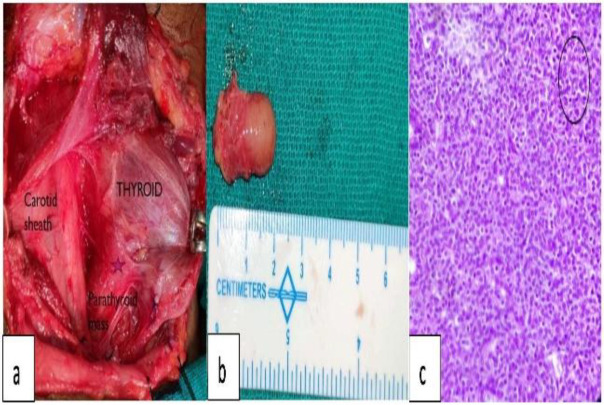
**(a) **Parathyroid mass identified at the inferior pole of the thyroid. **(b) **excised parathyroid specimen. **(c) **Histopathology showing chief cell hyperplasia suggesting parathyroid adenoma

Diagnosis of a parathyroid lesion with hyperparathyroidism with normocalcemic maxillary sinus Brown’s tumor was made. The patient was planned for right parathyroid mass excision. Intra-operatively, an enlarged right inferior parathyroid gland (figure 3a) was encountered. It was carefully separated from the thyroid gland and removed in toto. (figure 3b) Intraoperative intact parathyroid hormone level was assessed within 10 minutes post excision which dropped significantly from 297.7 pg/mL (preoperatively) to 9.7 pg/mL (reference range 15-65 pg/mL). Diagnosis of a parathyroid adenoma was concluded on histopathological examination (figure 3c).

**Fig 4:**
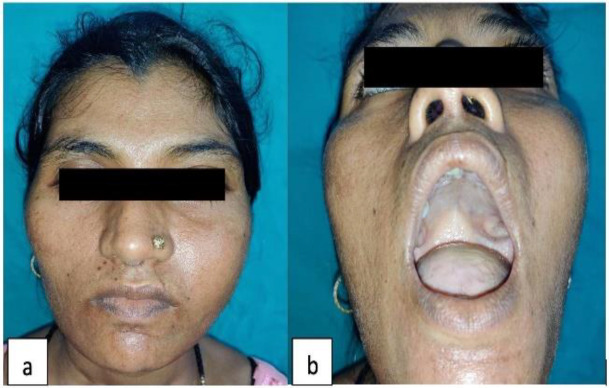
**(a) **Post-operative D7 **(b)** On 11 months follow-up visit with decreased palatal mass and decreased facial swelling

One day post-surgery patient developed hypocalcemia which was managed by calcium and Vitamin D supplements. The patient was kept under close observation for early detection of signs and symptoms of hypocalcemia for their timely management. Serial ECG monitoring followed by intravenous calcium gluconate administration was done when needed. The patient was discharged 7 days after surgery with near normal calcium and parathyroid hormone levels (29.39 pg/mL). Post-excision there was a significant reduction in the size of Brown’s tumor thus providing symptomatic relief to the patient (figure 4a,b). On 11 months follow-up, serum calcium, and parathyroid levels were found to be normal.

## Discussion

Brown’s tumor can be caused by primary, secondary, or tertiary hyperparathyroidism. Primary hyperparathyroidism is usually asymptomatic and its only initial manifestation is hypercalcemia (3). 

However rarely a patient can present with normal serum calcium level despite having a deranged parathyroid hormone level and associated skeletal lesions, as in our case. Our patient had no other signs and symptoms of hyperparathyroidism such as nephrocalcinosis, nephrolithiasis, or gastrointestinal and neurological manifestations and Brown’s tumor was the sole manifestation. So, in a patient with osteolytic bone lesions and raised parathyroid hormone assay, serum calcium levels can be normal and further workup should be done to rule out primary hyperparathyroidism. Brown’s tumor is usually seen in females of more than 50 years of age with the mandible being the most common site (5,6).

However, in our case, a 29-year female presented with maxillary sinus Brown’s tumor. Brown’s tumor can be misdiagnosed as a primary bone tumor on the basis of radiology, so cytology and blood investigations help to rule out other conditions such as aneurysmal bone cyst, osteosarcoma, giant cell reparative granuloma or giant cell tumor (9).

In a case report by Er et al., in a 57 year female with multiple sclerosis, a CT scan along with incisional biopsy, and biochemical investigations were done for diagnosing Brown’s tumor (10). However, in our case study, cytology of the lesion provided the much-needed diagnosis of Brown’s tumor thus avoiding the need for a biopsy from the maxilla. Moreover, cytology was helpful in supporting an inconclusive CT finding. Tc99m-sestamibi scan, which is a relatively newer radiological investigation, was not only confirmatory in diagnosis but also used for localizing the hyperfunctioning parathyroid gland. The most common cause of primary hyperparathyroidism is parathyroid adenoma which can be single or multiple (11). In our case the excised parathyroid mass, causing hyperparathyroidism came out to be a single right inferior parathyroid adenoma. Brown’s tumors regress on their own after parathyroidectomy and rarely may require surgical excision (12). 

In our patient following parathyroidectomy, serum PTH levels dropped to normal 10 minutes post excision. The patient’s serum calcium level also dropped which was corrected by giving calcium and vitamin D supplementation. The patient is now kept on regular follow-up and is symptomatically improving with an apparent decrease in size of the Brown’s tumors. On 11 months follow-up, the maxillary Brown’s tumor had more than 50% reduction in size with near normal appearance of the patient’s palate. Serum calcium and parathyroid levels were normal after 11 months of follow-up. 

## Conclusion

All osteolytic lesions of the maxilla and mandible do not require primary excision as the treatment of choice. Parathyroidectomy is the mainstay treatment for Brown’s tumor due to primary hyperparathyroidism.

Brown’s tumor regress after the management of parathyroid lesions. Even in the setting of normocalcemia, a patient presenting with locally aggressive osteolytic maxillary sinus mass, FNAC, and serum PTH levels should be done to rule out Brown’s tumor.
